# Assessing clinical trial failure risk factors and reasons in gastric cancer

**DOI:** 10.1186/s12876-022-02592-4

**Published:** 2022-11-30

**Authors:** Zikai Zhang, Junyi Yin, Yang Yue, Yang Su, Hong Jiang

**Affiliations:** 1 Department of Science, Tongji Hospital, Tongji University School of Medicine, Shanghai, 200092 China; 2 Department of Oncology, Tongji Hospital, Tongji University School of Medicine, Shanghai, 200092 China; 3 School of Mathematics and Statistics, University of Melbourne, Parkville, VIC 3010 Australia

**Keywords:** Clinical trials, Risk factor, Gastric cancer

## Abstract

**Background:**

Gastric cancer is one of the leading cancer-related death causes. Enormous efforts have been focused on this field in these years. However, clinical trial failure is becoming a massive obstacle for researchers to apply their research results for clinical use. This study aimed to analyze the reasons behind clinical failures and identify potential risk factors of clinical trial failures.

**Methods:**

On December, 1, 2021, we queried ClinicalTrials.gov for gastric cancer listed in phase II/III. We included trials specifying their interests in “stomach cancer”, “Stomach Neoplasms”, “Gastric Cancer”, “Gastric Neoplasms”, “Gastric Carcinoma”, “Stomach Carcinoma”, “Gastroesophageal Junction Cancer”. Exclude criteria are: (1) Trials that start prior to 01/01/2007 and start after 12/01/2020; (2) Trials with “not yet recruiting”, “suspended”, “withdrawn”, or “unknown” status; (3) Trials do not provide an anticipated accrual number or a start date.

**Results:**

A total of 567 trials are included. 10.2% of these trials are failed. 16 (2.82%) were terminated for good reasons, and 42 (7.41%) were terminated for bad reasons. Multi-centre (*P*-value = 0.088) and anticipated accrual (*P*-value = 0.099) are potential risk factors for clinical failures in the simple logistic regression model. After considering the interaction between multi-centre and anticipated accrual, the odds ratio of anticipated accrual is 0.60 (*P*-value = 0.009) in single centre trials. In multi-centre trials, the odds ratio of anticipated accrual is 0.72 (*P*-value = 0.025). The primary reason for gastric cancer trial terminations is recruitment failure.

**Conclusion:**

The rate that trials terminated in gastric cancer has decreased compared to previous studies. Comparing to other types of oncology trials, poor accrual continues to be the predominant reason, followed by business or sponsor reasons. Single-center trials with smaller anticipated accrual number are more likely to be terminated which may resulted by limited resources invested to the trial. Single-center design exacerbated the difficulty of participant recruitment. Future studies need to continue tracking the rate of trial termination across oncology and whether the reasons behind them have changed.

## Background

Gastric cancer is the third leading cause of cancer-related death globally [1]. In 2020, there are 1,089,103 new gastric cancer cases and 768,793 relevant deaths worldwide [2]. In the past decades, new therapeutical approaches to improve overall clinical outcomes have been developed and confirmed through a series of clinical trial phases and ensure both the safety and the efficacy according to the trial registration requirements of the United States Food and Drug Administration (FDA) and International Committee of Medical Journal Editors (ICMJE) [3, 4]. However, previous studies have highlighted that about 12% of those clinical trials were uncompleted, and that 28% of adult cancer trials were terminated with less than 90% projected subjects suffering from exaggerated symptoms described as an epidemic [5, 6]. Clinical trials that end early and do not follow previous study plans can hardly answer any research question. The New York Times criticized these failures as a waste of time and money and a massive obstacle to clinical researchers [7]. Indeed, even in the big data age, clinical trials still need carefully-planned and stable investments of time and funding.

Better understanding factors resulting in the failure of previous clinical trials can help avoid the recurrence of these failures in future research and decrease attrition in further clinical development [8]. Previous studies have studied clinical trial termination in diseases like cardiovascular diseases, urologic cancer, genitourinary cancer, and Alzheimer’s diseases, but clinical trial termination of gastric cancer is rarely reported [9–12].

To this end, gastric cancer trial data were hereby collected from ClinicalTrials.gov, and the rate of trial failure, the reasons for the failure, and estimated potential risk factors related to trial failure were then correspondingly analyzed. ClinicalTrials.gov is the largest clinical trial database worldwide that contains 400,873 research studies in all 50 states and 220 countries as of December 2021.

## Methods

### Data extraction and criteria

Data for gastric cancer listed in phase II/III were collected on December 1, 2021 by querying ClinicalTrials.gov. The search term included “Stomach Cancer”, “Stomach Neoplasms”, “Gastric Cancer”, “Gastric Neoplasms”, “Gastric Carcinoma”, “Stomach Carcinoma”, “Gastroesophageal Junction Cancer”. Some clinical trials may evaluate gastric cancer along with other types of cancers, we included them in our dataset.

**Fig. 1:**
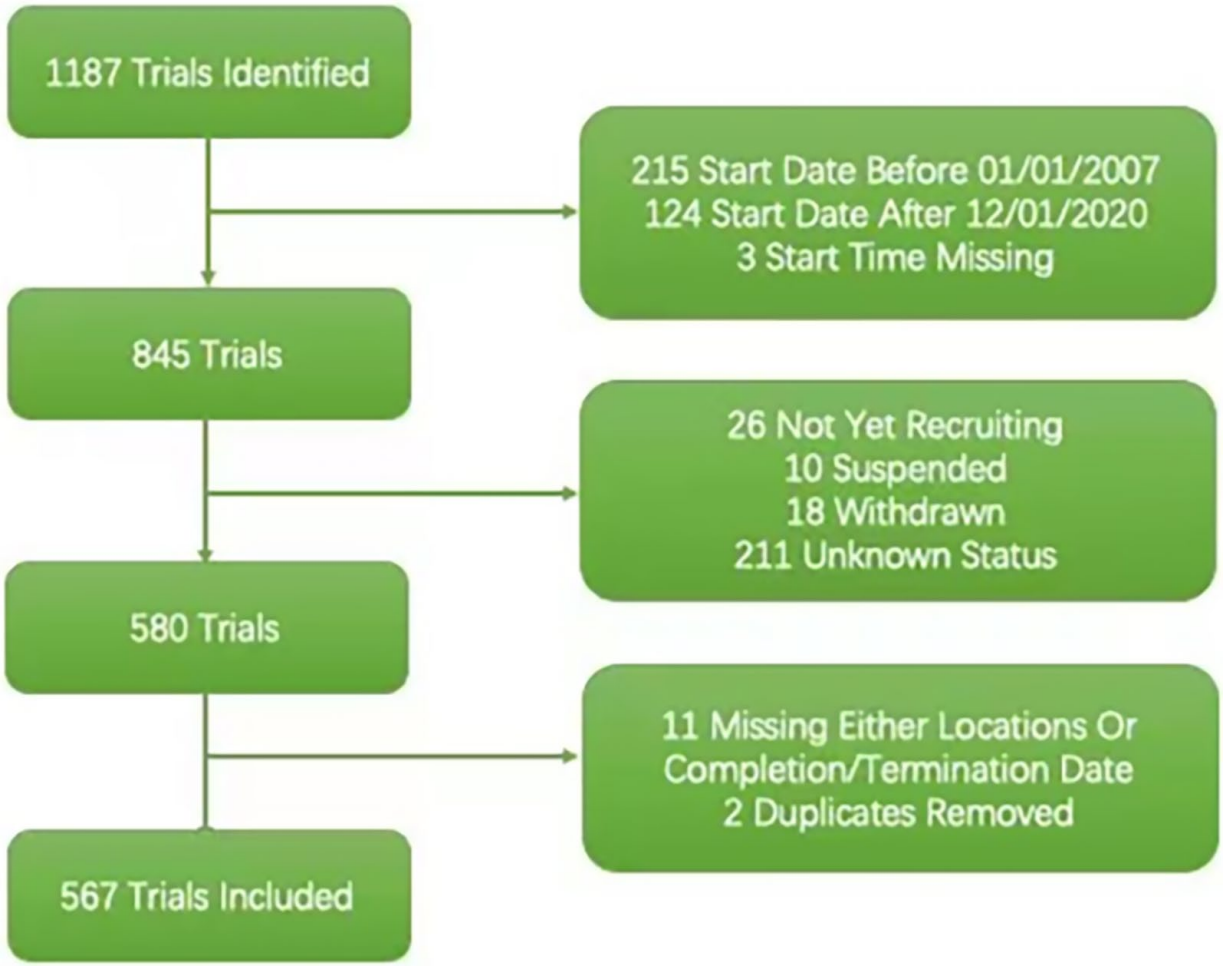
Data extraction protocol

According to the trial registration requirements of United States Food and Drug Administration (FDA) and International Committee of Medical Journal Editors (ICMJE), trials that started prior to January1, 2007 were excluded. We also excluded trials starting after 12/01/2020 to allow trials to leave at least 12 months for participants enrolling before the analysis. Trials at the status of “not yet recruiting”, “suspended”, “withdrawn” or “unknown” were ignored for unclear actual termination status. Trials that failed to provide an anticipated accrual number or a start date were also excluded from the dataset. Following these criteria, 567 trials were finally included in the dataset, and all clinical trials information and characteristic were downloaded and extracted from ClinicalTrials.gov XML files (Fig. 1).

### Data cleaning and trial characteristic classification

Clinical trial statuses of “recruiting”, “enrolling by invitation”, “active, not recruiting” were categorized as “active”. There is an optional text box enable trial managers to describe trial termination reason on ClinicalTrials.gov website since February 2007. Based on the information provided by this, two authors (JY and YS) separately classified them into the following nine reasons: safety reason, efficacy reason, ethical reason, trial no longer needed, business/sponsor reason, recruitment failure, logistic reason, PI left and no reason given. If two authors assign a trial into different categorizes, then a discussion will be made to determine which one is primary. Terminated trials attributed to factors including safety reason and efficacy reason were considered being terminated for good reasons, while those caused by factors like business/sponsor reason, ethical reasons, trials no longer needed, recruitment failure, logistic reason, PI left and no reason given were considered being terminated for bad reasons. Trials terminated for good reasons were hereby defined as a substantive outcome in the characteristic analysis of descriptive trials.

A trial involving more than one recruiting centre in its clinical site record was categorized it as a multi-centre trial; otherwise, a single-centre one. A trial involving multiple recruiting centres located in more than one country was labelled it as a multi-country clinical trial; otherwise, a single-country one. Besides, trials listed as phase I/II in the record were considered as phase II trials, while those listed as phase II/III in the record were phase III trials. It was found that the intervention type of some trials was mislabeled in the dataset. In this case, each trial was hereby reviewed by clinical experts and categorized according to its primary intervention type. In this study, each trial’s anticipated accrual number roughly followed a normal distribution, but some outliers in the trial anticipated accrual number were observed, as some phase II studies would enroll less than 10 participants, while some phase III studies would enroll more than 5,000 participants. To this end, the top 1% and bottom 1% of the data were winsorized. Since the distribution of anticipated accrual number is highly skewed, we log transformed this variable. Besides, the trial duration was calculated from the actual start date to the actual completion date or the termination date. For active studies, the trial duration was calculated from the actual start date to the date when the files were downloaded (Dec 1, 2021). Again, as extreme large phase III trials would consume significantly longer time than other trials, the top 1% of data were winsorized.

### Statistical analysis

The median anticipated trial accrual and trial duration were compared across studies using Kruskal-Wallis tests, and categorized variables like intervention type, phase, sponsor type were compared using chi-square tests.

Stata version 15 SE (Texas, USA) was used for comparing the risk factors and likelihood of clinical failures. Each trial’s duration was considered the survival time in our model, and characteristics in this study included phase, start year, treatment, sponsor type, single-centre versus multi-centre, single-country vs. multi-country, duration, anticipate accrual and status. Besides, logistic regression models were used to estimate how trial characteristics are correlated with clinical trial failure, and the cumulative Kaplan-Meier survival curve was adopted for the estimation of the failure risk of trials in different times.

## Results

**Fig. 2:**
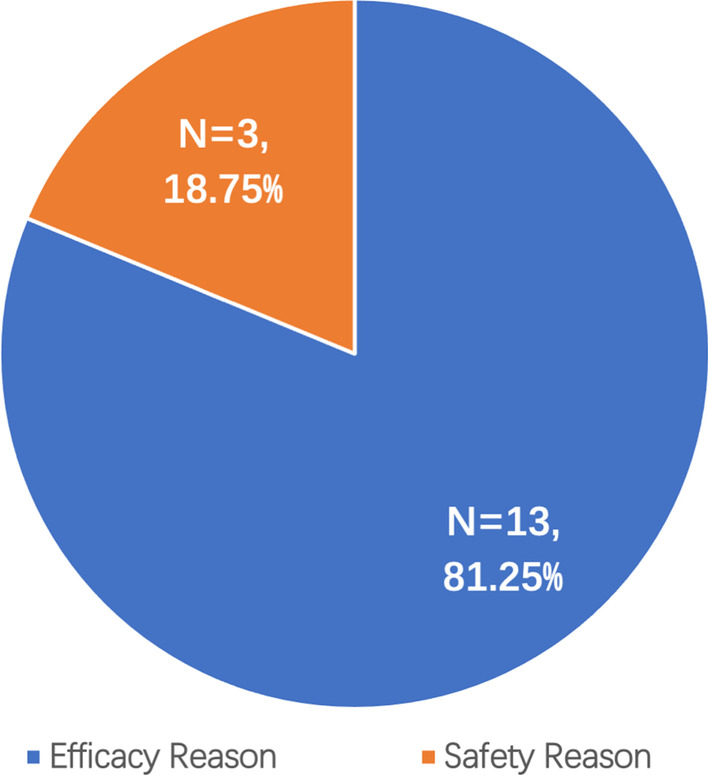
Reasons for gastric cancer trial terminated for good reasons

**Fig. 3:**
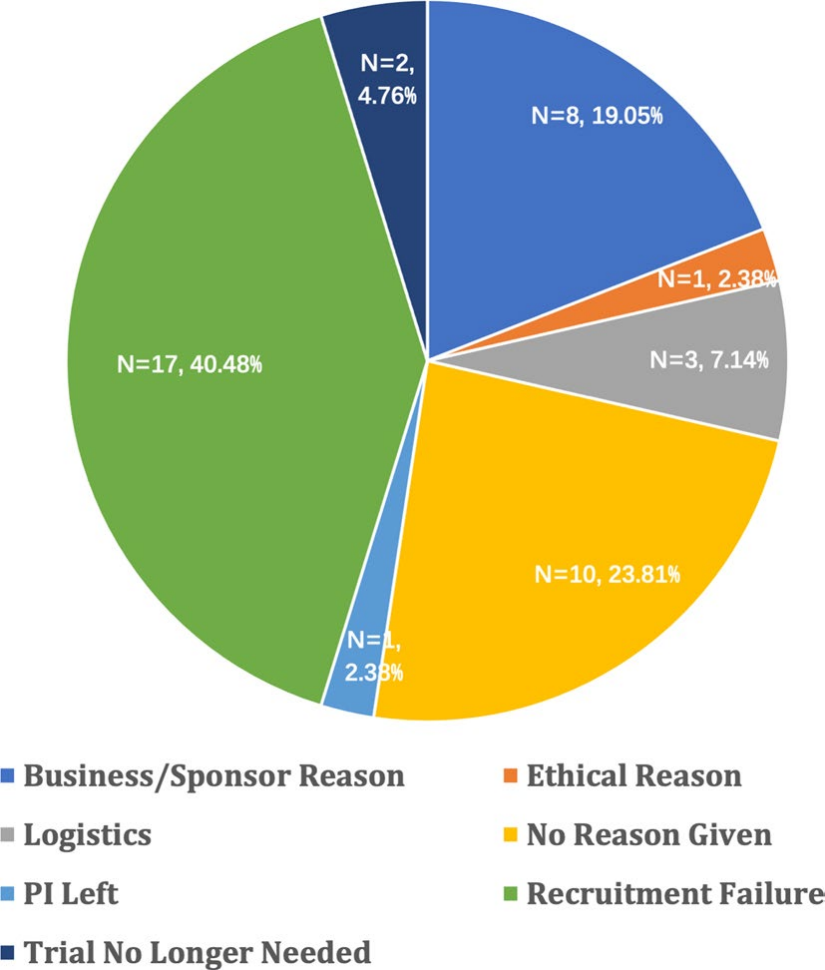
Reasons for gastric cancer trial terminated for bad reasons

**Fig. 4:**
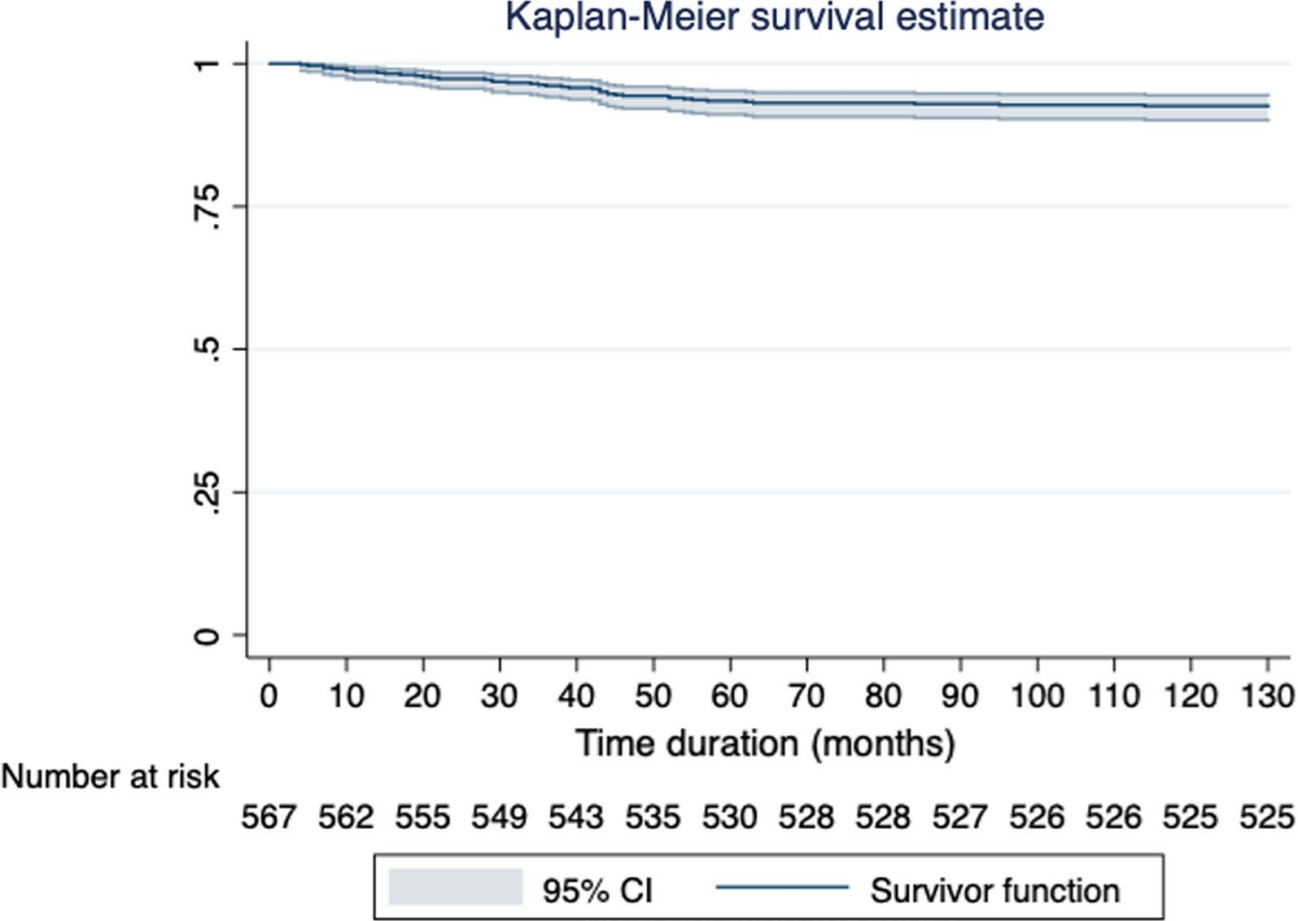
Cumulative incidence of trial failure

A total of 1187 clinical trials were identified, and 567 were included after extraction, among which, 263 (46.38%) were active, 246 (43.39%) were completed, 16 (2.82%) were terminated for good reasons, and 42 (7.41%) were terminated for bad reasons. Among trials terminated due to good reasons, 13 (81.25%) were terminated for efficacy reasons; 3 (18.75%) for safety reasons (Fig. 2). Among trials terminated due to bad reasons, 17 (40.48%) trials were terminated due to recruitment failure, which is the major problem of trial failure; 10 (23.81%) trials were terminated without giving a reason; 8 (19.05%) trials were terminated due to business or sponsor reasons; 3 (7.7%) trials were terminated due to logistic reasons such as the unavailability of drugs; 2 (4.76%) trials were terminated for the outcome of other studies has already provided enough information for clinical interests, and the trial is no longer needed; 1 (2.38%) trial was terminated for ethical reason and 1 (2.6%) trial was terminated due to PI leaving the institution (Fig. 3). Almost every one in ten gastric cancer trials (10.2%) were terminated. The cumulative Kaplan-Meier survival estimate is illustrated in Fig. 4.

The descriptive characteristics analysis of this study is illustrated in Table 1. It was found that 74.07% of gastric cancer trials were phase II trials. And, there was no evidence supporting that the percentage of phase II and III was differential among different statuses (*P*-value = 0.463). Indeed, most trials that started before 2010 have been completed now, while one large phase III trial in 2007 and one large phase III trial in 2008 are still active. Besides, trials that started in 2011 and 2012 were more likely to be terminated before completion, and active trials accounted for the highest percentage of genetic treatment, indicating the popularity of immunology as a hot research spot in these years.

**Table 1:** Descriptive characteristics of clinical trials

	Failed (N = 42)	Completed (N = 247)	Active (N = 262)	Good terminated (N = 16)	*P*-value
Phase					0.463
Phase II	33 (78.57%)	184 (74.49%)	192 (73.38%)	11 (68.75%)	
Phase III	9 (21.43%)	63 (25.51%)	70 (26.72%)	5 (31.25%)	
Start year					< 0.001
2007	2 (4.76%)	25 (10.12%)	1 (0.38%)	1 (6.25%)	
2008	3 (7.14%)	31 (12.55%)	1 (0.38%)	1 (6.25%)	
2009	4 (9.52%)	28 (11.34%)	1 (0.38%)	0	
2010	2 (4.76%)	23 (9.31%)	1 (0.38%)	0	
2011	5 (11.90%)	28 (11.34%)	2 (0.76%)	3 (18.75%)	
2012	7 (16.67%)	27 (10.93%)	2 (0.76%)	3 (18.75%)	
2013	4 (9.52%)	21 (8.50%)	11 (4.20%)	2 (12.50%)	
2014	3 (7.14%)	17 (6.88%)	7 (2.67%)	3 (18.75%)	
2015	4 (9.52%)	22 (8.91%)	12 (4.58%)	0	
2016	1 (2.38%)	3 (1.21%)	23 (8.78%)	0	
2017	5 (11.90%)	14 (5.67%)	38 (14.50%)	2 (12.50%)	
2018	2 (4.76%)	6 (2.43%)	43 (16.41%)	1 (6.25%)	
2019	0	2 (0.81%)	59 (22.52%)	0	
2020	0	0	61 (23.28%)	0	
Treatment					< 0.001
Drug	30 (71.43%)	186 (75.30%)	153 (58.40%)	14 (87.50%)	
Device	4 (9.52%)	20 (8.10%)	19 (7.25%)	0	
Genetic	8 (19.05%)	32 (12.96%)	71 (27.10%)	1 (6.25%)	
Radiation	0	8 (3.24%)	8 (3.05%)	1 (6.25%)	
Other	0	1 (0.4%)	11 (4.20%)	0	
Sponsor					0.356
NIH	2 (4.76%)	21 (8.54%)	11 (4.20%)	0	
Industry	20 (47.62%)	106 (42.91%)	125 (47.71%)	6 (37.50%)	
Other	20 (47.62%)	120 (48.58%)	126 (48.09%)	10 (62.50%)	
Multi-centre					0.062
No	15 (35.71%)	132 (53.44%)	123 (46.95%)	5 (31.25%)	
Yes	27 (64.29%)	115 (46.56%)	139 (53.05%)	11 (68.75%)	
Multi-country					0.093
No	29 (69.05%)	205 (83.00%)	202 (77.10%)	11 (68.75%)	
Yes	13 (30.95%)	42 (17.00%)	60 (23.90%)	5 (31.25%)	
Anticipated accrual	123.21 (132.80)	159.67 (202.20)	237.57 (268.70)	209.94 (207.19)	0.001
Duration	36.36 (24.34)	46.48 (23.41)	43.40 (26.10)	34.38 (15.25)	0.363

Moderate evidence was found in the simple logistic regression model that trials conducted in multi-centre are 1.77 (*P*-value = 0.088) times more likely to be terminated than those conducted in a single centre. The odds ratio of trial anticipated accrual number is 0.78 (*P*-value = 0.099), indicating that anticipated accrual number is a weak protective risk factor for clinical trial termination. Besides, there was no strong or overwhelming evidence supporting that the trial phase, sponsor type, treatment type and multi-country are potential risk factors of clinical trial failure (Table 2).

**Table 2:** Univariate logistic regression model

Covariates	Odds ratio	95% CI	*P*-value
Phase			
Phase II	Reference		
Phase III	0.76	(0.36, 1.64)	0.491
Treatment			
Drug	Reference		
Device	1.21	(0.41, 3.61)	0.736
Genetic	0.91	(0.40, 2.03)	0.809
Radiation	Omit		
Other	Omit		
Sponsor			
Other	Reference		
NIH	0.80	(0.18, 3.58)	0.771
Industry	1.08	(0.57, 2.06)	0.815
Multi-centre			
No	Reference		
Yes	1.77	(0.92, 3.39)	0.088
Multi-country			
No	Reference		
Yes	1.75	(0.88, 3.48)	0.110
Anticipated accrual	0.78	(0.58, 1.05)	0.099

Small clinical trials are considered more likely to be designed as single-centre trials. Thus, an interaction between anticipated accrual and multi-centre was hereby included in the final multiple logistic regression model. As illustrated in Table 3, for single center trials, the odds ratio of anticipating accrual number is 0.60 (*P*-value = 0.009), indicating that trials initially planned to enroll less participant will increase risk of trial termination. For multi-center trials, the odds ratio of anticipating accrual number is 0.72 (0.60 × 1.21 = 0.72, *P*-value = 0.025). Therefore, the effect of increasing anticipating accrual number to prevent clinical trial failure is weaker in multi-center trials.

**Table 3:** Multiple logistic regression model

Covariates	Odds ratio	95% CI	*P*-value
Anticipated accrual	0.60	(0.41, 0.88)	0.009
Anticipated accrual# Multi-centre			
No	Reference		
Yes	1.21	(1.02, 1.42)	0.025

## Discussion

Clinical trial is an essential part of clinical research. It is estimated that 10.2% of gastric cancer trials are terminated before reaching endpoints. Compared to the clinical trial failure rate in other cancer types, that in gastric cancer is higher than that of clinical failure in all cancer types [5, 13]. There is a decrease from 12% back in 2015, which may be related to more well-designed trials in recent years. It can be observed from the cumulative incidence graph that the failure rate is relatively stable after 50 months, and this phenomenon can be explained as the likeliness of trial failure in their early periods. Besides, recruitment failure is the primary cause of clinical trial termination, which is consistent across all kinds of oncology in previous studies [5, 8, 10, 14]. In order to improve enrollment in clinical trials, several studies have made recommendations to improve accrual rate such as taking measurements to better inform participants about the program ; when a patient is a good candidate for clinical trials and there is no trial available in the institution, considering search trials in other institutions; re-examine eligibility criteria; reducing participants’ travel burden [14–16].

Our study also finds that the trial with a larger anticipated accrual number design is more likely to be completed, and it is believed that more resources and funding from clinical trial sponsors are invested in large trials. Besides, possibly, trial sponsors have a higher expectation that these trials can answer their vital clinical research questions. Another potential reason is that large trials may expand their inclusion criteria and lengthen the recruiting period. Multi-centre is considered to have an interaction with the anticipated accrual number. Early studies reported multi-centre or anticipated accrual number as a risk factor, and this is the first time attempts are made to combine the effects of recruiting in multi-centre and anticipate accrual number [10, 17, 18]. Recruiting participants in multiple centers increases the possibility of clinical trials enrolling more participants, while adding funding to single sites is proven not the reason that leads to a contemporaneous increase in trial recruitment [19]. However, choosing the right trial sites is a potential challenge for multicenter studies, which increases the risk of trial failure. It is still found after adding this interaction in the hereby proposed model that adding more anticipants can completely cancel the multicenter effect. In other words, if a single-centre study fails to satisfy the accrual goal during future gastric cancer trial planning, the advantages of choosing multiple centres to allow more participants are overwhelming. Business or sponsor reasons are often correlated to recruitment failure. For example, the limited resources that the sponsor invested on the trial may restricted the number of recruiting sites which may lead to accrual failure.

It should be also noted that trial phase, treatment type, treatment type and multi-country are not potential risk factors in this study. In these years, massive efforts have been made on participant protection and trial design, and FDA has updated several versions of data monitoring requirements and clinical study design guidelines in past decades. Thus, participants are now more willing to be enrolled in different clinical trials, while sponsors are less likely to stop funding the trial once the trial starts. Also, the fasting growing field of genetic inhibitors may also relate to this finding. According to a previous study, only around 8% of trials included genetic interventions, and the industry-sponsored only accounted for 34% in 2011–2015 [20]. In our study, genetic interventions are increased to approximately 20%, and 45.1% of these trials are sponsored by the industry. There is no doubt that genetic treatments like PD-1 inhibitors etc. are hot spots in contemporary clinical research, and that industry companies are now investing huge time and money in this field.

This study is based on the assumption that failed or terminated trials for bad reasons fail to provide essential information for clinical trials. However, whether these trials can provide the researcher with some information needs further investigation. Many trials recruiting about 80–85% of the participants they need can still answer their clinical questions. Additionally, 10 terminated trials are found terminated without a specific reason, and the research evidence may become more solid if this very reason can be clarified.

## Conclusion

The rate that trials terminated in gastric cancer has decreased compared to previous studies. Comparing to other types of oncology trials, poor accrual continues to be the predominant reason, followed by business or sponsor reasons. Single-center trials with smaller anticipated accrual number are more likely to be terminated which may resulted by limited resources invested to the trial. Single-center design exacerbated the difficulty of participant recruitment. Future studies need to continue tracking the rate of trial termination across oncology and whether the reasons behind them have changed.

## Data Availability

The datasets used and analyzed during the current study are available from the corresponding author on reasonable request.

## Abbreviations

FDA: Food and Drug Administration
ICMJE: International Committee of Medical Journal Editors
