# Correction: Assessing clinical trial failure risk factors and reasons in gastric cancer

**DOI:** 10.1186/s12876-023-02642-5

**Published:** 2023-02-09

**Authors:** Zikai Zhang, Junyi Yin, Yang Yue, Yang Su, Hong Jiang

**Affiliations:** 1 Department of Science, Tongji Hospital, Tongji University School of Medicine, Shanghai, 200092 China; 2 Department of Oncology, Tongji Hospital, Tongji University School of Medicine, Shanghai, 200092 China; 3 School of Mathematics and Statistics, University of Melbourne, Parkville, VIC 3010 Australia

**Correction to: BMC Gastroenterology (2022) 22:496** **https://doi.org/10.1186/s12876-022-02592-4**

After publication of this article [1], the authors reported that in the Funding information section the following was missing: ‘Grants from the Foundation of Shanghai Municipal Health Commission (Grant No. 201740038)’.

The original article [1] has been corrected.
